# Moderately prolonged dry intervals between precipitation events promote production in *Leymus chinensis* in a semi-arid grassland of Northeast China

**DOI:** 10.1186/s12870-021-02920-y

**Published:** 2021-03-20

**Authors:** Jinwei Zhang, Xiangjin Shen, Bifan Mu, Yujie Shi, Yuheng Yang, Xuefeng Wu, Chunsheng Mu, Junfeng Wang

**Affiliations:** 1 Key Laboratory of Vegetation Ecology, Ministry of Education, Institute of Grassland Science, School of Life Sciences, Northeast Normal University, Changchun, 130024 P.R. China; 2 Northeast Institute of Geography and Agroecology, Chinese Academy of Sciences, Changchun, 130102 P.R. China; 3 School of Life Sciences, Northeast Normal University, Changchun, 130024 P.R. China

**Keywords:** Climate change, Rainfall patterns, Grassland productivity, Soil nitrogen, Drought stress

## Abstract

**Background:**

Climate change is predicted to lead to changes in the amount and distribution of precipitation during the growing seasonal. This “repackaging” of rainfall could be particularly important for grassland productivity. Here, we designed a two-factor full factorial experiment (three levels of precipitation amount and six levels of dry intervals) to investigate the effect of precipitation patterns on biomass production in *Leymus chinensis* (Trin.) Tzvel. (a dominant species in the Eastern Eurasian Steppe).

**Results:**

Our results showed that increased amounts of rainfall with prolonged dry intervals promoted biomass production in *L. chinensis* by increasing soil moisture, except for the longest dry interval (21 days). However, prolonged dry intervals with increased amount of precipitation per event decreased the available soil nitrogen content, especially the soil NO_3_^−^-N content. For small with more frequent rainfall events pattern, *L. chinensis* biomass decreased due to smaller plant size (plant height) and fewer ramets. Under large quantities of rain falling during a few events, the reduction in biomass was not only affected by decreasing plant individual size and lower ramet number but also by withering of aboveground parts, which resulted from both lower soil water content and lower NO_3_^−^-N content.

**Conclusion:**

Our study suggests that prolonged dry intervals between rainfall combined with large precipitation events will dramatically change grassland productivity in the future. For certain combinations of prolonged dry intervals and increased amounts of intervening rainfall, semi-arid grassland productivity may improve. However, this rainfall pattern may accelerate the loss of available soil nitrogen. Under extremely prolonged dry intervals, the periods between precipitation events exceeded the soil moisture recharge interval, the available soil moisture became fully depleted, and plant growth ceased. This implies that changes in the seasonal distribution of rainfall due to climate change could have a major impact on grassland productivity.

**Supplementary Information:**

The online version contains supplementary material available at 10.1186/s12870-021-02920-y.

## Background

Water availability is the primary constraint to plant productivity in arid and semi-arid ecosystems [1, 2], and it will be strongly affected by ongoing and future climate change [3]. The availability of soil water for plants is regulated by both the amount and distribution of precipitation [1–3]. Currently, intensive research has revealed that grassland productivity is strongly positively correlated with the amount of precipitation [1, 4]. However, for a given site, the relationship is not always linearly correlated [5, 6] because of the variation in the distribution of precipitation events within the growing season [7].

Climate change scenarios predict significant alterations in the amount and distribution of precipitation in arid and semiarid ecosystems, which may result in changes to plant productivity [8, 9]. In general, small and tightly clustered precipitation events evaporate rapidly and only wet the surface soil, which exerts a limited effect on plant productivity [10]. However, it can promote productivity in shallow-rooted plants, such as bunchgrass [11]. Intermediate intervals between moderate precipitation events may trigger a series of shorter periods of biomass production as the soil begins to dry between these events; however, the available soil moisture will not become fully depleted unless the interval between precipitation events exceeds the soil moisture recharge interval. When the available soil water supply becomes fully depleted, biomass production ceases [12, 13]. When larger precipitation events occur, the deeper penetration of soil water into the profile and the lower proportional loss to evaporation increases the amount and duration of water in the soil for plant uptake [1, 14–16]. In addition to being limited by soil moisture, soil nutrient content is inherently low in arid ecosystems and potentially limiting to plant growth [13, 17]. Precipitation can directly influence soil nutrients through leaching and runoff. Frequent large rainfall events will increase the potential for loss of soil nutrients through leaching, with the nutrients accumulating in deep soil layers below the rooting zone in arid ecosystems [18, 19]. Meanwhile, soil nutrients can also be indirectly affected by vegetation absorption [20, 21]. Therefore, the responses of plant growth to changes in rainfall patterns may be affected by both soil water and nutrient availability [7, 13, 17, 22].

*Leymus chinensis* (Trin.) Tzvel. is a dominant perennial rhizome grass widely distributed in the Eastern Eurasian Steppe from North Korea to Mongolia and Northern China and north-westward to Siberia [23]. This grassland area is about 4.2 × 10^5^ km^2^, and *L. chinensis* accounts for 80–90% of the grassland’s productivity [24]. In addition to its wide distribution and high yield, *L. chinensis* also has high forage quality (19.5% crude protein, 3.1% crude fat, 35% crude fiber, and 6% ash) for cattle and sheep [25]. The change in *L. chinensis* yield is closely related to local ecosystem health and livestock production [26, 27]. As a typical semi-arid grassland, *L. chinensis* grassland productivity is regulated by precipitation patterns [4, 26, 28]. Previous research has revealed that the possible reasons for the variations in *L. chinensis* grassland productivity caused by the amount of rainfall are shifts in the size of individual plants size or density of *L. chinensis* [4]. However, the influence that the dry intervals between rainfall events on *L. chinensis* grassland productivity is still unclear.

Rainfall patterns are currently changing and are predicted to continue changing with global warming [3]. A deeper understanding of the impacts of rainfall amounts and intervals on dominant species is essential for predicting grassland productivity due to future variation in rainfall patterns. In this study, experiments were carried out to investigate the response of *L. chinensis* to changes in long-term averages as well as inter-annual variation in rainfall amounts and intervals. The first objective of this study was to quantify how *L. chinensis* growth varied in response to precipitation pattern changes. Because the effects of precipitation patterns on plant growth are likely due to the direct effects of altered soil moisture and nutrients, the second objective of this study was to investigate the underlying mechanisms of *L. chinensis* growth responses to precipitation pattern changes by quantifying the effects of soil moisture and nutrients on plant growth. Based on the above research we propose the two hypotheses. 1) Small and tightly clustered precipitation events only wet the surface soil and evaporate rapidly, and exhibit a limited effect on plant productivity. However, moderately prolonged intervals with larger rainfall events will enhance soil moisture in both shallow and deep soil layers, and will further improve plant growth. 2) Under conditions of extremely prolonged dry intervals, soil moisture becomes a fully depleted factor because the very low levels of moisture in the soil between rainfall events exceed plant tolerance, and lead to plant growth ceasing.

## Results

### Effects of rainfall treatments on plant biomass production and allocation

**Fig. 1:**
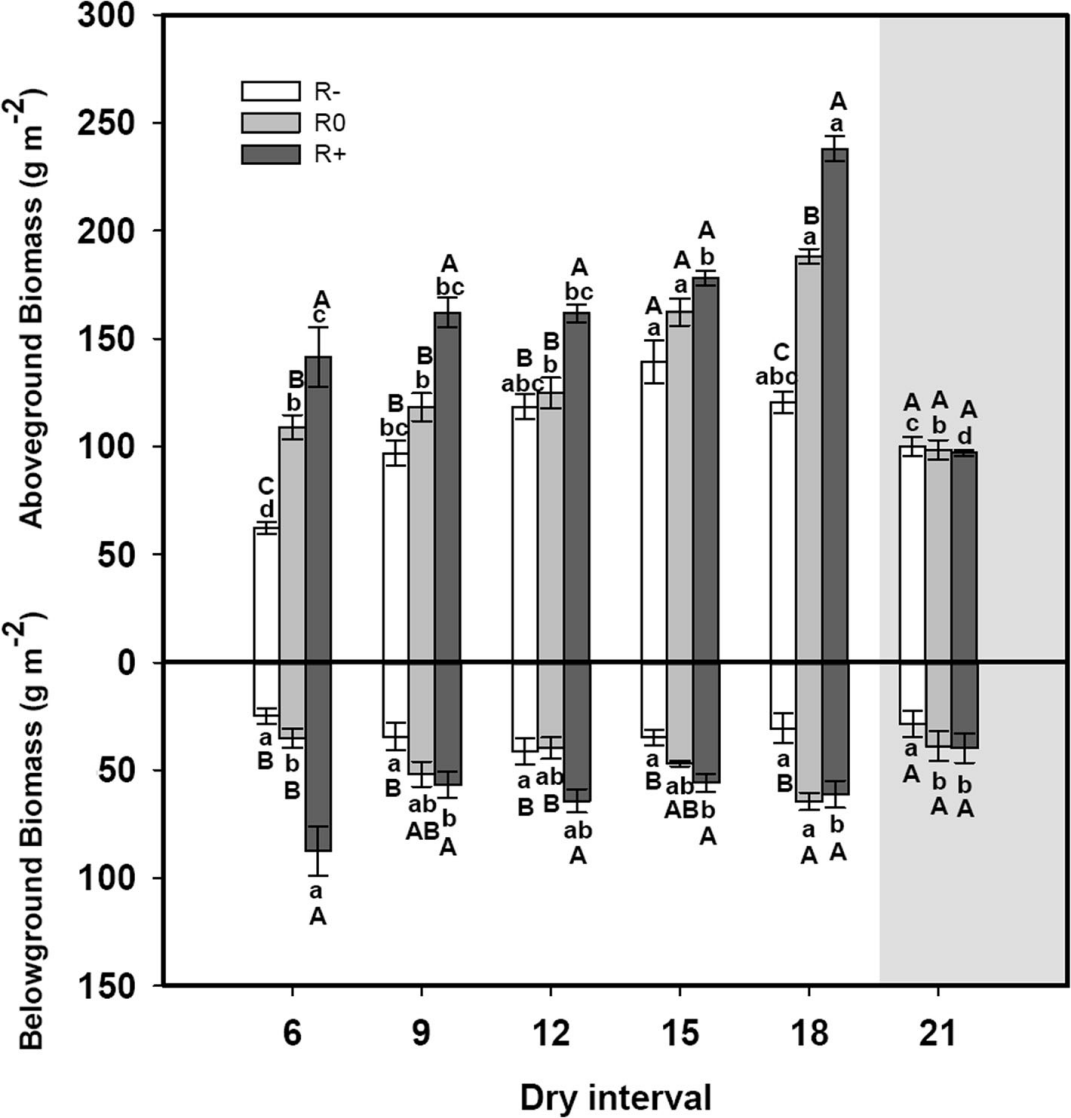
Responses of *L. chinensis* aboveground biomass and belowground biomass to the variation in rainfall amounts and dry intervals. The values at each dry interval are the means ± SE (*n* = 4). Capital letters indicate significant differences (*p* < 0.05) between rainfall amount treatments and small letters between dry interval treatments. *L. chinensis* withered during the 21-day interval (shadowed section)

Changes in the amount of rainfall and the length of the dry intervals had significant main effects on aboveground biomass and belowground biomass (Table 1). With increases in rainfall amounts, aboveground biomass and belowground biomass significantly increased, except for the 21 days dry interval treatment (Fig. 1). As the length of the dry intervals extended, aboveground biomass and belowground biomass showed a monopeak curve (Fig. 1). The maximum values of aboveground biomass were 139 g m^− 2^ under the 15 days dry interval treatment for R-, and 188 g m^− 2^ and 237 g m^− 2^ for R0 and R+, respectively, under the 18 days dry interval treatment (Fig. 1). Under the 21 days dry interval treatment, the aboveground parts of *L. chinensis* were withered after about 60 days of treatment (at late July) (Fig. 1). In addition, changes in the rainfall amount and dry interval also had significant interactive effects on biomass production (Table 1).

**Table 1:** Results (F-values) of two-way ANOVAs for the effects of rainfall amount and dry interval on plant traits and soil properties

	Amount		Interval		Amount×Interval	
	F	*p*	F	*p*	F	*p*
Plant traits						
Height	42.81	**< 0.001**	20.91	**< 0.001**	3.47	**0.001**
Number of ramets	9.95	**< 0.001**	9.33	**< 0.001**	1.17	0.330
Aboveground biomass	120.69	**< 0.001**	77.62	**< 0.001**	11.12	**< 0.001**
Belowground biomass	34.87	**< 0.001**	2.79	**0.026**	4.25	**< 0.001**
R/S	4.00	**0.024**	5.00	**0.001**	2.28	**0.026**
S/L	8.39	**0.001**	22.18	**< 0.001**	2.93	**0.005**
Soil properties						
NH_4_^+^-N content	6.44	**0.003**	8.48	**< 0.001**	0.34	0.964
NO_3_^−^-N content	51.08	**< 0.001**	77.93	**< 0.001**	3.99	**< 0.001**
Available phosphorus	3.33	**0.043**	5.67	**< 0.001**	0.40	0.940
Soil water content	3971.40	**< 0.001**	3230.97	**< 0.001**	74.44	**< 0.001**

Note: Bold values are significant at *p* < 0.05

**Fig. 2:**
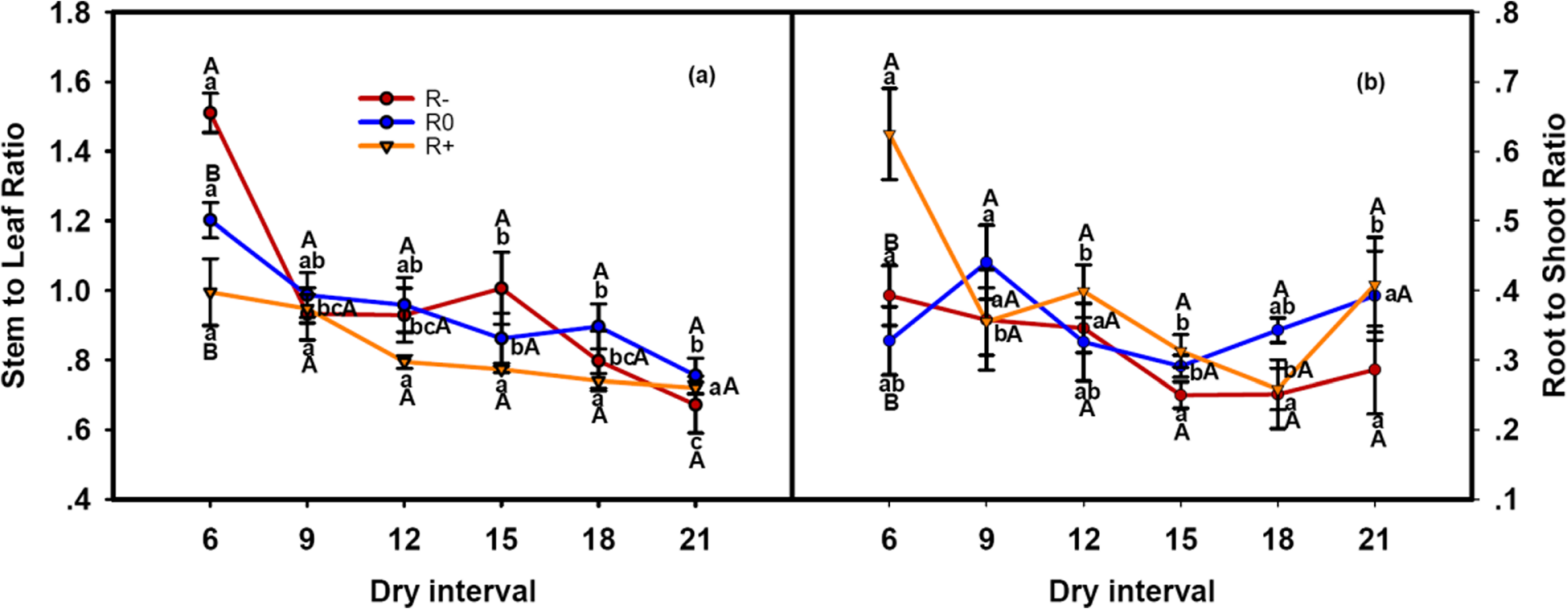
Responses of (**a**) the ratio of stem to leaf and (**b**) the ratio of root to shoot to the variation in rainfall amounts and dry intervals. The values at each dry interval are the means ± SE (*n* = 4). Capital letters indicate significant differences (*p* < 0.05) between rainfall amount treatments and small letters between dry interval treatments

Changes in the rainfall amount and dry interval and their interaction had significant effects on the S/L and R/S of *L. chinensis* (Table 1). Increased amounts of rainfall and extended dry intervals significantly lowered S/L (Fig. 2a). Under prolonged dry intervals, the R/S values produced a concave curve (Fig. 2b).

### Effects of rainfall treatments on plant height and ramet number

**Fig. 3:**
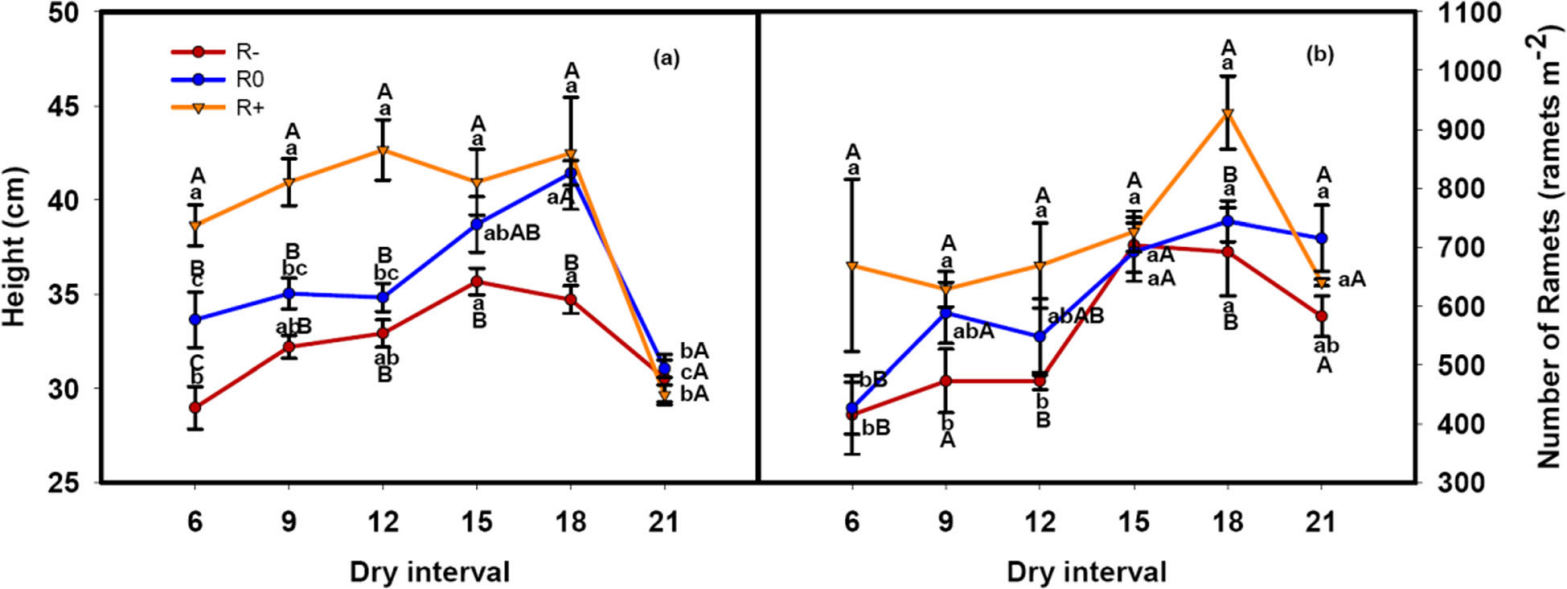
Responses of (**a**) plant height and (**b**) ramets number to the variation in rainfall amounts and dry intervals. The values at each dry interval are the means ± SE (*n* = 4). Capital letters indicate significant differences (p < 0.05) between rainfall amount treatments and small letters between dry interval treatments

Changes in the rainfall amount and dry interval had significant main effects on the plant height and ramet number (Table 1). Increases in the amount of rainfall led to a significant increase in plant height and ramet number except for the D21 treatment (Fig. 3a, b). With extension of the dry intervals, plant height and ramet number increased initially, but then decreased (Fig. 3a, b). For R-, peak values for plant height and ramet number were observed under the D15 treatment, with the maximum values reached for R0 and R+ with the D18 treatment (Fig. 3a, b). Changes in the amount of rainfall amount and the dry interval also had significant interactive effects on plant height (Table 1).

### Effects of rainfall treatments on soil properties

**Fig. 4:**
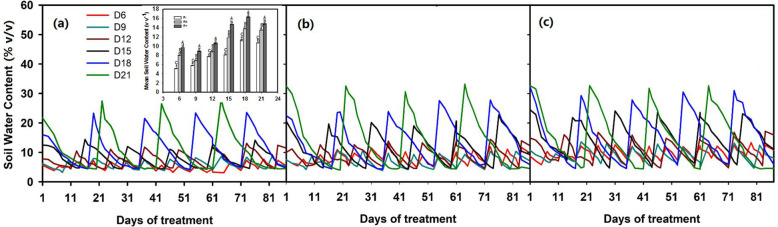
The dynamics of soil water content in pots during the experimental period. **a**, **b** and **c** represent decreased (− 30%), control and increased (+ 30%) rainfall amounts, respectively. The different colored lines represent different rainfall intervals. The inset indicates the mean soil water content (MSWC) under different rainfall amounts and dry interval treatments. Small letters indicate significant differences in MSWC between rainfall intervals

Changes in the amount of rainfall, the length of dry intervals, and their interaction had significant effects on mean soil water content (MSWC) (Table 1). Increased rainfall amounts resulted in significant increases in the MSWC (Fig. 4a, b, c). With prolonged dry intervals, the MSWC first increased and then decreased (Fig. 4a, b, c). The maximum MSWC values were observed in the D18 treatment, reaching 11.3 (% v v^− 1^), 13.7 (% v v^− 1^), and 16.3 (% v v^− 1^) for R-, R0, and R+, respectively (Fig. 4a).

**Fig. 5:**
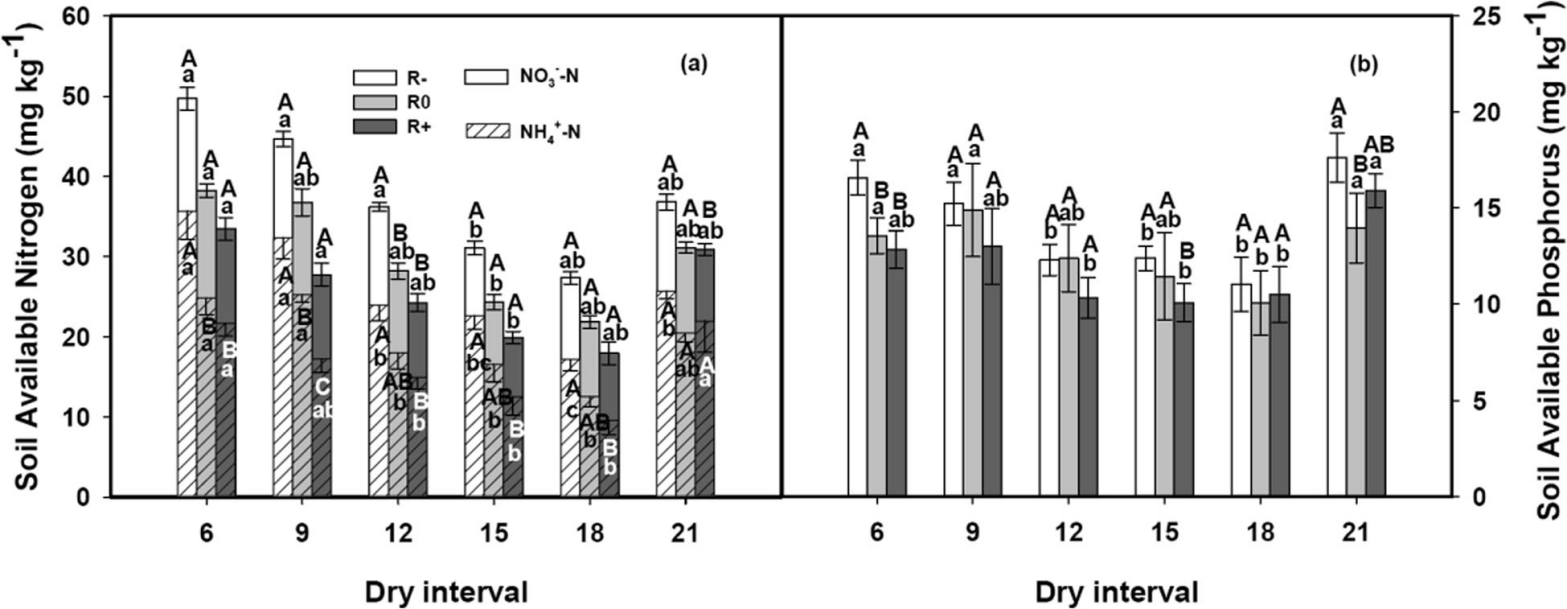
Responses of (**a**) soil nitrate nitrogen content (NO3^-^-N) and soil ammonium nitrogen content (NH4 ^+^ -N), and (**b**) available soil phosphorus to the variation in rainfall amounts and dry intervals. The values at each dry interval are the means ± SE (*n* = 4). Capital letters indicate significant differences (p < 0.05) between rainfall amount treatments and small letters between dry interval treatments

Regarding available soil nutrients, changes in the rainfall amount and the dry interval length had significant main effects on the available soil NH_4_^+^-N, NO_3_^−^-N, and P (Table 1). Elevated rainfall decreased the available soil NH_4_^+^-N, NO_3_^−^-N, and P (Table 1, Fig. 5a, b), but with prolonged dry intervals the trend was an initial reduction and then a rise (Fig. 5a, b). Minimum values of available soil N and P were observed under the D18 treatment for R-, R0, and R+ (Fig. 5a, b). Furthermore, changes in the rainfall amounts and dry intervals also had significant interactive effects on NO_3_^−^-N (Table 1).

### The response mechanism of plant biomass production to variation in rainfall patterns

**Fig. 6:**
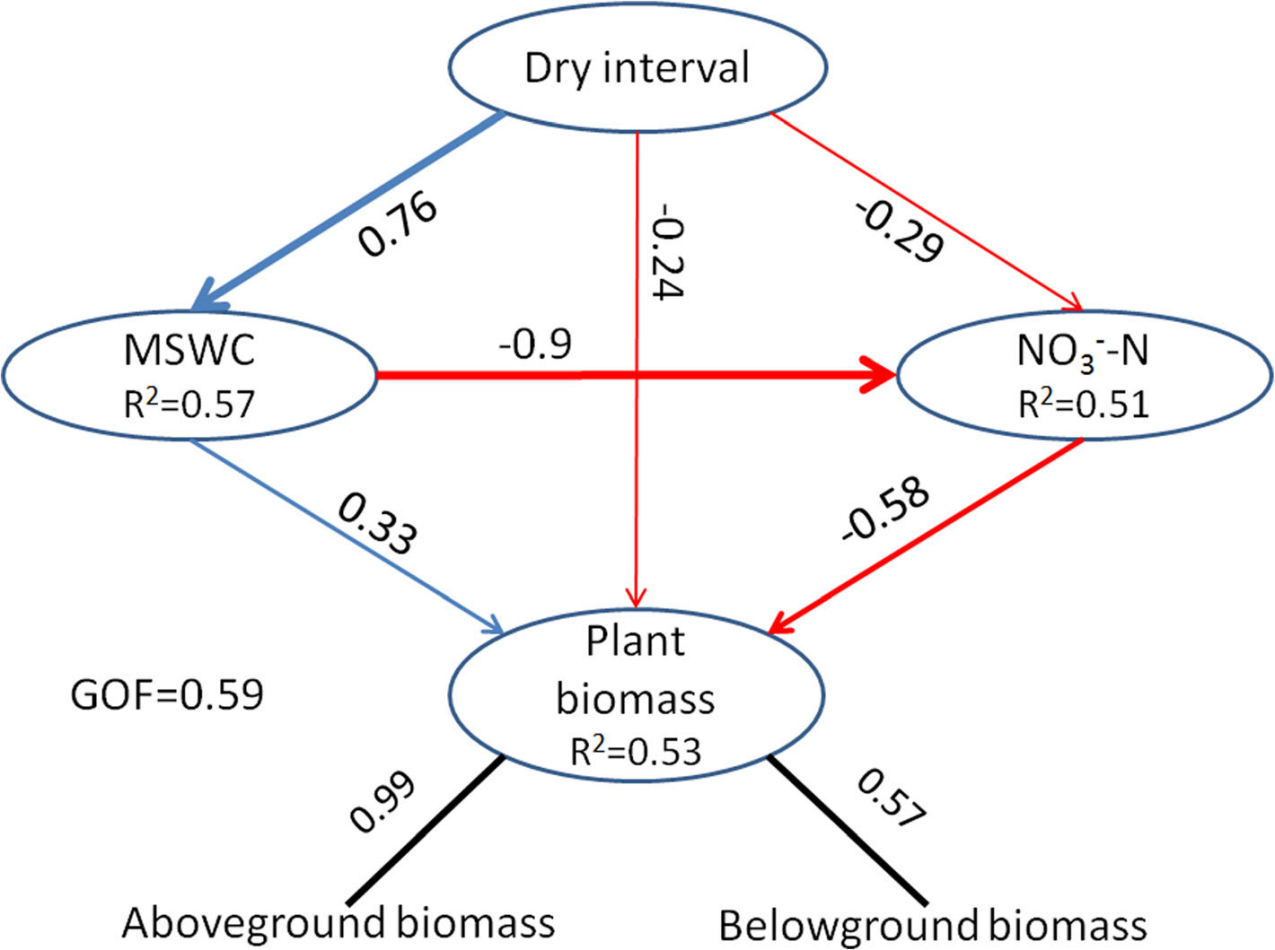
The direct and indirect effects of dry interval, mean soil water content and soil NO3^-^-N on plant biomass were determined by partial least squares path modeling. Observed variables are represented in the ellipses. The loadings (the correlations between observed variables) are indicated as the values near the arrowed line, and the path coefficients (between observed variables) and the coefficients of determination (R^2^ in ellipses) were calculated after 999 bootstraps. The model was assessed using the goodness of fit (GOF) statistic, a measure of the overall prediction performance. The GOF was 0.59

Before undertaking PLS-PM (partial least squares path modeling) analysis, a stepwise regression method was used to determine the relative importance of environmental factors (rainfall amounts, dry intervals, available soil NH_4_^+^-N, NO_3_^−^-N, P, and MSWC) in explaining plant biomass. We found that dry intervals, MSWC, and soil NO_3_^−^-N significantly influenced plant biomass. The subsequent PLS-PM illustrated the direct and indirect relationships of plant biomass production to dry intervals, MSWC, and NO_3_^−^-N. The dry intervals showed a direct negative (effect size − 0.24) relationship to biomass production. Dry intervals can also influence biomass production by altering MSWC (effect size 0.76) and NO_3_^−^-N (effect size − 0.29). The MSWC exhibited a positive effect on plant biomass (effect size 0.33), and a negative effect on NO_3_^−^-N (effect size − 0.9). NO_3_^−^-N showed a negative relationship to biomass production (effect size − 0.58) (Fig. 6).

## Discussion

Except for the 21-day interval treatment, the plant aboveground biomass and belowground biomass increased notably with increasing amounts of rainfall, irrespective of either short or long dry-interval treatments (Fig. 1). In fact, it has been established previously that elevated amounts of rainfall can alleviate drought in arid and semi-arid ecosystems [29, 30]. The reason for the positive correlation between biomass production and moderately prolonged dry intervals between rainfall events is likely due to an increase in soil moisture at depths where evaporative demand is negligible (deep in the 0-10 cm profile), and this would contrast with situations where small amounts of precipitation of water deeper into the profile [15] (Fig. 4). Indeed, the fine roots of *L. chinensis* were located in the 0-10 cm soil profile, and so the plant can utilize both shallow and deeper soil moisture resources [31]. The plant water status under different rainfall patterns also supported the results of aboveground biomass changing (Fig. S2). The plant aboveground parts withered in the late-July, the net photosynthetic rate and transpiration rate were zero. After rewatering on 2nd Aug, the new ramets were germination from the underground bud. The new leaves had high photosynthetic rate and transpiration rate, so the average photosynthetic rate and transpiration rate in 21-day dry interval were no significant different even higher than 6-day dry interval (Fig. S2 a and b). However, the green leaves to total leaves ratio reached the minimum value in 21-day interval treatment (Fig. S2 d). This result is partly similar to the study conducted in perennial *Bouteloua gracilis* grassland where fewer but larger rainfall events produced the highest value of aboveground biomass in northeastern Colorado [15]. It also supports our first hypothesis that prolonged dry intervals combined with large rainfall events can improve plant productivity in semi-arid grassland. However, our result is inconsistent with research on the effects of dry intervals on the growth of *Agriophyllum squarrosum* in the Mu Us Desert [29]. Firstly, this could be due to the soil texture in the Mu Us Desert is sandy and characterized by lower water-holding capacity [29]. However, the soil in our study site is clay loam, which has higher water-holding capacity than sandy soil [32]. Secondly, *A. squarrosum* is an annual forb, has a different response to soil moisture circumstance [15, 29]. Thus, it suggests that the response of vegetation to rainfall pattern varies with soil types and species changes [33].

Under low rainfall amount combined with more frequent rainfall events, *L. chinensis* aboveground biomass reduced due to smaller plant size and lower ramet numbers (Fig. 1, Fig. 3). Meanwhile, higher values for the S/L and R/S ratios were observed (Fig. 2). These results indicate that *L. chinensis* can adopt drought avoidance strategies to minimize water loss and maintain plant tissue hydration during such rain patterns. However, if the dry interval is too long and exceeds the plant’s drought duration tolerance, aboveground parts of the plant may wither (Fig. 1, Fig. 3). The mechanisms for this phenomenon include loss of antioxidant defenses in chloroplasts, and accelerating senescence in aboveground tissues to protect the belowground buds from water loss [34, 35]. Plants can resist and survive drought through different adaptive strategies, but there will be a decline in plant productivity. This result confirmed the second hypothesis that once the interval between precipitation events exceeds the soil moisture recharge interval, plant growth ceases.

Previous studies have shown that the amount of annual rainfall only partially explains differences in grassland productivity [36]. The length of the intervals between rainfall events and the intensity of the precipitation can also be identified as important regulators of plant growth [37]. In our study, we found that dry intervals can influence plant biomass production by altering the MSWC and available soil nitrogen (Fig. 6). In addition, the available soil nitrogen content decreased following prolonged the intervals between rainfall events (Fig. 5). It is possible that prolonged dry intervals combined with large rainfall events for a given amount of rainfall can increase soil moisture content, and then enhance the solubility of available soil nutrients. Then, the soil available nutrient could be more easily absorbed by plants [20]. Furthermore, increases in rainfall intensity per event can lead to leaching of available nitrogen into deeper soil layers [18], and high water content also can increase denitrification rates by displacing oxygen and creating anoxic conditions in the soil, which then stimulate anaerobic reduction of nitrate to N_2_ and N_2_O [21]. It is suggested that increasing frequencies of heavy precipitation events due to climate change may accelerate the loss of available soil nitrogen in semi-arid grassland ecosystems in the future.

## Conclusions

Our results indicate that: 1) for certain combinations of prolonged dry intervals and intervening high rainfall events, semi-arid grassland productivity might be improved, but these conditions may lead to nitrogen limitation via leaching and plant absorption. 2) Under extremely prolonged dry intervals, even though the mean soil water content may be high, when the length of the dry period exceeds the soil moisture recharge interval, plant growth ceases. The yield of *L. chinensis* directly affects the livestock production and local economy of Eurasian grassland. Meanwhile, *L. chinensis* is a typical perennial rhizomatous grass, the response of *L. chinensis* to the rainfall pattern variation can also reflect the response of other perennial grass, at least rhizomatous grass, to the rainfall pattern changes. These results imply that moderately prolonged dry intervals with intervening large precipitation events can promote semi-arid grassland productivity, but extremely prolonged dry intervals restrict production. This study highlights the crucial role that altered patterns of precipitation could have on grassland productivity in semi-arid ecosystems.

## Methods

### Study site and plant material

This experiment was conducted at the central Songnen grassland (Songnen Grassland Ecological Research Station of Northeast Normal University, Changling County in Jilin Province, China, 44°45′N, 123°45′E), located on the Eastern Eurasian Steppe. This part of grassland belongs to the experimental land of the Northeast Normal University for the research of grassland science, and the university allows us to experiment here. This area has a typical mesothermal monsoon climate with dry and cold winters and relatively wet and warm summers [32, 38]. Annual mean temperature ranges from 4.6 °C to 6.4 °C and annual precipitation ranges from 280 to 400 mm with about 80% of precipitation events having occurred during the period from June to August in the past 50 years (1961–2010). The mono-dominated species is *Leymus. chinensis* (Trin.) Tzvel. (Herbarium of Northwest A & F University (WUK, 0442655)) in this study area. *L. chinensis* is a perennial clonal plant with vigorous belowground rhizomes. It mainly relies on vegetative propagation for population renewal and has high palatability for livestock such as cattle and sheep [39]. Therefore, to predict the production of *L. chinensis*, it is necessary to understand the influence of rainfall variation on its growth in this area.

### Experiment design and field manipulation

We used a two-factor randomized complete block design to manipulate the amounts of precipitation and dry intervals between precipitation events from June to August 2018. According to historical rainfall data at the local site (data from Changling County Meteorological Bureau), three levels of controlled precipitation were used. The long-term average biologically effective precipitation during the period from June 1st to September 1st was 334 mm (R0), and a 30% decrease and a 30% increase relative to the long-term average biologically effective rainfall of 233 mm and 434 mm (R- and R+), respectively, were also used. Events with daily precipitation greater than or equal to 2 mm were regarded as biologically effective events [15]. Meanwhile, cases of more than three days of consecutive precipitation were divided into two events [15]. At this site, the dry intervals between rainfall events ranged from 8.6 days to 13.3 days. Based on IPCC predictions that the dry intervals between rainfall events will be prolonged in the future [9], we selected six levels of dry intervals; six days, nine days, twelve days, fifteen days, eighteen days, and twenty-one days.

To determine rainfall amounts and intervals more accurately, we conducted a simulated rainfall experiment under an arched rainout shelter with steel frames and clear polyethylene roofs (the length and width of the shelter were 6.5 m and 4.5 m, respectively). On May 15, 2018, Professor Chunsheng Mu undertook the formal identification of the plant material and chose a patch of homogeneous *L. chinensis* grassland for experiment. We removed the litter, then dug out plant-soil cores (each 24 cm in diameter and 25 cm in depth) from the homogeneous *L. chinensis* grassland. There were 18 (3 × 6) treatments with four replicates each, totaling 72 (18 × 4) plant-soil cores for this study. After being dug out carefully, each of the plant-soil cores was transferred to a plastic pot (24 cm diameter and 26 cm height). The rooting system of *L. chinensis* is mainly distributed within the 0-10 cm soil depth, so the height of the pot is sufficient for plant root growth [31, 40]. Before the experiment, plants were allowed to acclimatize to their pots for 15 days, and were watered adequately every three days (a total of five times) to ensure survival and even growth (about 11.4 ± 1.3 cm high for plant in each pot), and the values of soil water content (measured with a TRIME Pico64 (IMKO. GmbH. Ettlingen. Germany)) were similar in each pot (6.03 ± 0.52% v v^− 1^). The volume of experimental water was quantified with a measuring cylinder, and we used a watering can to simulate natural precipitation, with watering occurring between 6:30 and 9:30 AM (for detailed information about the intensity and intervals of the rainfall events see Table 2).

**Table 2:** The detailed date and intensity for each rainfall event. The frequencies for the 6-day, 9-day, 12-day, 15-day, 18-day and 21-day rainfall intervals were 15, 10, 8, 6, 5 and 4, respectively. The total rainfall amounts for the R-, R0 and R+ treatments were 233 mm, 334 mm and 434 mm, respectively

		5/31	6/6	6/9	6/12	6/15	6/18	6/21	6/24	6/27	6/30	7/6	7/12	7/15	7/18	7/24	7/30	8/2	8/5	8/11	8/14	8/17	8/20	8/23
R-	D6	15.5	15.5	\	15.5	\	15.5	\	15.5	\	15.5	15.5	15.5	\	15.5	15.5	15.5	\	15.5	15.5	\	15.5	\	15.5
	D9	23.3	\	23.3	\	\	23.3	\	\	23.3	\	23.3	\	23.3	\	23.3	\	23.3	\	23.3	\	\	23.3	\
	D12	33.3	\	\	33.3	\	\	\	33.3	\	\	33.3	\	\	33.3	\	33.3	\	\	33.3	\	\	\	33.3
	D15	38.9	\	\	\	38.9	\	\	\	\	38.9	\	\	38.9	\	\	38.9	\	\	\	38.9	\	\	\
	D18	46.7	\	\	\	\	46.7	\	\	\	\	46.7	\	\	\	46.7	\	\	\	46.7	\	\	\	\
	D21	58.4	\	\	\	\	\	58.4	\	\	\	\	58.4	\	\	\	\	58.4	\	\	\	\	\	\
R0	D6	22.2	22.2	\	22.2	\	22.2	\	22.2	\	22.2	22.2	22.2	\	22.2	22.2	22.2	\	22.2	22.2	\	22.2	\	22.2
	D9	33.3	\	33.3	\	\	33.3	\	\	33.3	\	33.3	\	33.3	\	33.3	\	33.3	\	33.3	\	\	33.3	\
	D12	47.6	\	\	47.6	\	\	\	47.6	\	\	47.6	\	\	47.6	\	47.6	\	\	47.6	\	\	\	47.6
	D15	55.6	\	\	\	55.6	\	\	\	\	55.6	\	\	55.6	\	\	55.6	\	\	\	55.6	\	\	\
	D18	66.7	\	\	\	\	66.7	\	\	\	\	66.7	\	\	\	66.7	\	\	\	66.7	\	\	\	\
	D21	83.4	\	\	\	\	\	83.4	\	\	\	\	83.4	\	\	\	\	83.4	\	\	\	\	\	\
R+	D6	28.9	28.9	\	28.9	\	28.9	\	28.9	\	28.9	28.9	28.9	\	28.9	28.9	28.9	\	28.9	28.9	\	28.9	\	28.9
	D9	43.3	\	43.3	\	\	43.3	\	\	43.3	\	43.3	\	43.3	\	43.3	\	43.3	\	43.3	\	\	43.3	\
	D12	61.9	\	\	61.9	\	\	\	61.9	\	\	61.9	\	\	61.9	\	61.9	\	\	61.9	\	\	\	61.9
	D15	72.3	\	\	\	72.3	\	\	\	\	72.3	\	\	72.3	\	\	72.3	\	\	\	72.3	\	\	\
	D18	86.7	\	\	\	\	86.7	\	\	\	\	86.7	\	\	\	86.7	\	\	\	86.7	\	\	\	\
	D21	108.4	\	\	\	\	\	108.4	\	\	\	\	108.4	\	\	\	\	108.4	\	\	\	\	\	\

In our experiment, the arrangement of pots is shown in Fig. S1. The pots were placed 0.75 m away from the edge of shelter to prevent exposure from ambient rainfall. The aisles between the treatments were 0.5 m. The distance between pots in a row was 0.13 m. Final harvest was on the 26th of August 2018, and the time elapsed from the start was 87 days. To simulate natural conditions, 72 pots were buried into the soil with the upper edge 1 cm above soil surface. The shelter’s roof was only used during rain events, so once the weather turned clear we removed the polyethylene roof immediately. Each pot had a 1.5-cm diameter hole in the bottom to allow for drainage [28].

### Soil properties

For each pot, the soil volume water content at the depth of 10 cm was measured with a TRIME Pico64 (IMKO. GmbH. Ettlingen. Germany) field moisture TDR-sensor between 16:00–17:00 pm every one to two days. The mean soil water content (MSWC) was calculated as the averaged value of soil water content during the experiment. At the end of the treatment, three soil cores (diameter 2 cm, depth 25 cm) were taken from each pot (carefully removing the plant material) and were mixed in sealed bags to form one composite sample. The soil samples were kept in a cooler during transport to the laboratory where they were carefully homogenized and sieved through 2 mm mesh. Each sample was separated into two parts after the removal of roots. One part was kept at − 20 °C to measure soil ammonium nitrogen content (NH_4_^+^-N) and nitrate nitrogen content (NO_3_^−^-N). The remainder was used to measure the available P concentration after being air-dried for 15-days. The concentrations of soil NH_4_^+^-N and NO_3_^−^-N were determined using a continuous flow analyzer (Alliance Flow Analyzer, Futura, Frépillon, France). Soil available P was determined via the molybdate blue colorimetric method following extraction with 0.5 mol L^− 1^ NaHCO_3_.

### Plant performance

Three plants of per pot were randomly marked to measure the photosynthetic index. The plant net photosynthetic rate (A), transpiration rate (E), water use efficiency (WUE) were measured with an open gas-exchange system (PPsystem, CIRAS-3, Hasha Scientific Instruments Limited, USA). In order to reduce the experiment error, the photosynthetic indexes were measured on the day before and after watering respectively between 1st Aug to 15th Aug. The average of the two measurements represents the actual photosynthetic index. Meanwhile, we observed and recorded the green leaves to total leaves ratio per pot on the 25th of August 2018.

We collected the above- and below-ground parts (washed free of soil) of plants for each pot at the end of the experiment. Individual plant heights were measured and the number of ramets was counted for every pot. Belowground parts were gently washed of soil and collected with a 1 mm mesh sieve. The plant leaves, stems, and belowground parts of each pot were detached and weighed separately after drying at 65 °C for 48 h. Aboveground biomass was calculated as the sum of the dry masses of leaves and stems. Biomass allocation explained how plants allocate their biomass to different organs [41]. The Stem/Leaf ratio (S/L) was determined as the ratio of stem dry biomass to leaf dry biomass, which can be calculated with the following equation [41]:

1 $$ \mathrm{S}/\mathrm{L}=\frac{\mathrm{Stem}\ \mathrm{dry}\ \mathrm{mass}\ \left(\mathrm{g}\ {\mathrm{m}}^{-2}\right)\ }{\mathrm{Leaf}\ \mathrm{dry}\ \mathrm{mass}\ \left(\mathrm{g}\ {\mathrm{m}}^{-2}\right)} $$

The Root/Shoot ratio (R/S) can be calculated with the following equation [41]:

2 $$ \mathrm{R}/\mathrm{S}=\frac{\mathrm{Belowground}\ \mathrm{dry}\ \mathrm{mass}\ \left(\mathrm{g}\ {\mathrm{m}}^{-2}\right)}{\mathrm{Aboveground}\ \mathrm{dry}\ \mathrm{mass}\ \left(\mathrm{g}\ {\mathrm{m}}^{-2}\right)} $$

### Statistical analysis

We used two-way ANOVAs to assess the effects of rainfall amounts and intervals and their interaction with soil properties (soil available P content, NH_4_^+^-N content, NO_3_^−^-N content, and MSWC) and vegetation performance (plant height, number of ramets, aboveground biomass, belowground biomass, S/L, and R/S). Differences between treatments were compared by Duncan’s multiple range tests. The above analyses were performed using SPSS 21.0 statistical software (SPSS Institute, Cary, NC, U.S.A.). Partial least squares path modeling (PLS-PM, using the inner plot function in the R plspm package) was used to further identify the possible pathways by environment factors to control for *L. chinensis* biomass accumulation. Before PLS-PM, the stepwise regression method was used to determine the relative importance of environmental factors (rainfall amount, dry interval, NH_4_^+^-N, NO_3_^-.^-N, soil available P, and MSWC) in explaining plant biomass.

## Supplementary Information

**Additional file 1.**

## Data Availability

The dataset supporting the conclusions of this article is included within the supplementary file (Electronic supplementary material-data.xlsx). It is available from the corresponding author on reasonable request.

## Abbreviations

R0: The long-term average biologically effective rainfall amount during the period from June 1st to September 1st
R +: a 30% increase relative to the long-term average biologically effective rainfall amount during the period from June 1st to September 1st
R-: a 30% decrease relative to the long-term average biologically effective rainfall amount during the period from June 1st to September 1st
D6: Six days dry intervals between rainfall events
D9: Nine days dry intervals between rainfall events
D12: Twelve days dry intervals between rainfall events
D15: Fifteen days dry intervals between rainfall events
D18: Eighteen days dry intervals between rainfall events
D21: Twenty-one days dry intervals between rainfall events
MSWC: The mean soil water content
NH_4_^+^-N: Soil ammonium nitrogen content
NO_3_^−^-N: Soil nitrate nitrogen content
P: Phosphorus
S/L: The Stem/Leaf ratio
R/S: The Root/Shoot ratio
PLS-PM: Partial least squares path modeling
