# Supplementary material for: Moderately prolonged dry intervals between precipitation events promote production in Leymus chinensis in a semi-arid grassland of Northeast China
Source: BMC Plant Biol. 2021 Mar 20;21:147. doi: 10.1186/s12870-021-02920-y (PMC7981859; doi:10.1186/s12870-021-02920-y)
Supplement: Supplementary file 1 — Additional file 1. [file 12870_2021_2920_MOESM1_ESM.docx]

Supplemental Material


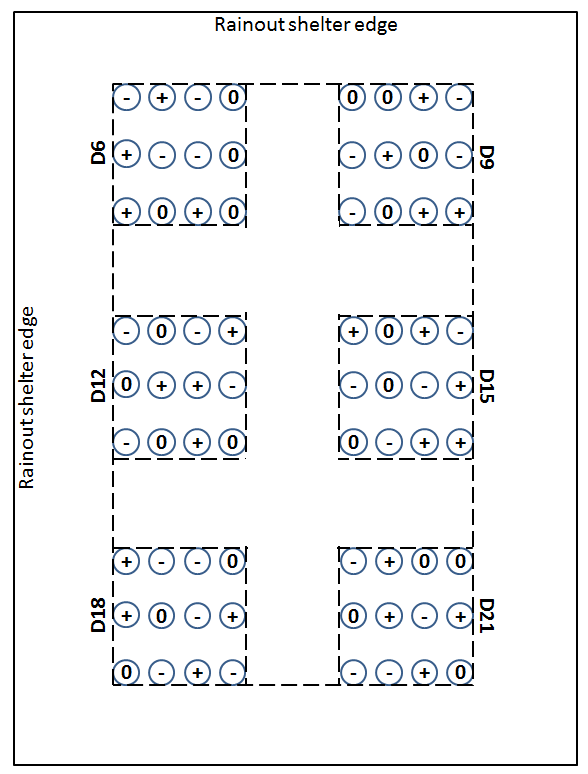


Figure S1. Schematic illustration of the overall experimental design, including three levels of rainfall amounts (average rainfall amount: R0 (0); 30% increased and decreased rainfall amount: R+ (+) and R- (-)) and six levels of dry intervals (D6: 6-day interval; D9: 9-day interval; D12: 12-day interval; D15: 15-day interval; D18: 18-day interval; D21: 21-day interval).


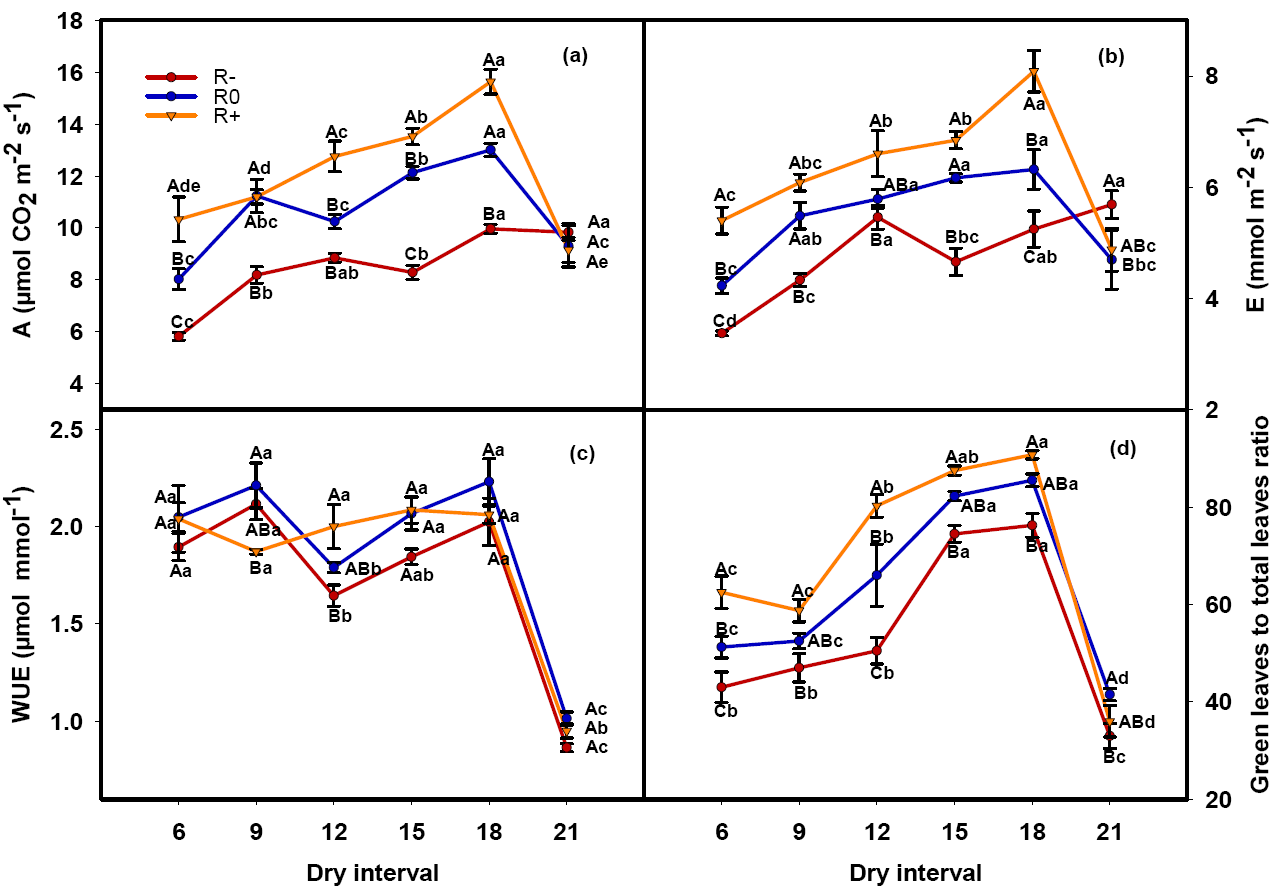


Figure S2. Responses of (a) the net photosynthetic rate (A), (b) the transpiration rate (E), (c) water use efficiency (WUE) and green leaves to total leaves ratio (d) to the variation in rainfall amounts and dry intervals. The values at each dry interval are the means ± SE (n=4). Capital letters indicate significant differences (*p*<0.05) between rainfall amount treatments and small letters between dry interval treatments.

Table S1. Total amounts and intensity of rainfall events for treatments in the experiment.

| Total amounts | | Event size (mm) for each dry interval | | | | | |
| --- | --- | --- | --- | --- | --- | --- | --- |
| % change | mm | 6-day | 9-day | 12-day | 15-day | 18-day | 21-day |
| 70 | 233 | 15.5 | 23.3 | 33.3 | 38.9 | 46.7 | 58.4 |
| 100 | 334 | 22.2 | 33.3 | 47.6 | 55.6 | 66.7 | 83.4 |
| 130 | 434 | 28.9 | 43.3 | 61.9 | 72.3 | 86.7 | 108.4 |

Table S2. Results (F-values) of two-way ANOVAs for the effects of plant net photosynthetic rate (A), transpiration rate (E), water use efficiency (WUE) and green leaves to total leaves ratio on *Leymus chinensis*.

|  | Amount | | Interval | | Amount×Interval | |
| --- | --- | --- | --- | --- | --- | --- |
|  | F | *p* | F | *p* | F | *p* |
| A | 101.66 | **<0.001** | 41.46 | **<0.001** | 8.02 | **<0.001** |
| E | 45.89 | **<0.001** | 23.78 | **<0.001** | 5.93 | **<0.001** |
| WUE | 5.92 | **0.005** | 85.75 | **<0.001** | 1.96 | 0.06 |
| Green leaves to total leaves ratio | 50.1 | **<0.001** | 144.19 | **<0.001** | 3.22 | **0.003** |

Note: Bold values are significant at *p*<0.05.
